# Thermal Stability, Optical and Electrical Properties of Substoichiometric Molybdenum Oxide

**DOI:** 10.3390/ma16072841

**Published:** 2023-04-02

**Authors:** Yubin Qing, Kaijun Yang, Yaofeng Chen, Jinpeng Zhu, Yujing Li, Chong Chen, Qingkui Li, Benshuang Sun, Jilin He

**Affiliations:** 1 School of Materials Science and Engineering, Zhengzhou University, Zhengzhou 450001, China; 2 Zhongyuan Critical Metals Laboratory, Zhengzhou University, Zhengzhou 450001, China; 3 Central China Branch, Oriental Green Energy (Hebei) Co., Ltd., Zhengzhou 450003, China

**Keywords:** substoichiometric molybdenum oxide, functional ceramics, thermal stability, decomposition mechanism, reflectivity, resistivity

## Abstract

Substoichiometric molybdenum oxide ceramics have aroused widespread interest owing to their promising optical and electrical performance. In this work, the thermal stability and decomposition mechanism of Mo_9_O_26_ and Mo_4_O_11_ at 700–1000 °C and 700–1100 °C were investigated, respectively. Based on this information, MoO_x_ (2 < x < 3) bulk ceramics were prepared by spark plasma sintering (SPS). The results show that Mo_9_O_26_ is stable up to 790 °C in an argon atmosphere. As the temperature rises, it decomposes into Mo_4_O_11_. Mo_4_O_11_ can exist stably at 830 °C, beyond which it will convert to MoO_2_. The MoO_x_ ceramic bulks with four different components (MoO_2.9_, MoO_2.8_, MoO_2.7_ and MoO_2.6_) were successfully sintered by SPS, and their relative density was greater than 96.4% as measured by the Archimedes principle. The reflectivity of MoO_x_ ceramic bulk is low and only 6.3% when the composition is MoO_2.8_. The resistivity increases from 10^−3^ to 10^−1^ Ωcm with the increase in the O/Mo atomic ratio x. In general, the thermal stability information provides a theoretical basis for the processing of MoO_x_ materials, such as the sintering of the MoO_x_ target. The optical and electrical properties show that MoO_x_ is a low-reflective conductive oxide material with great photoelectric application value.

## 1. Introduction

Molybdenum oxide comes in various forms, including molybdenum trioxide (MoO_3_) with a wide gap (>2.7 eV), substoichiometric molybdenum oxide (MoO_x_) with oxygen vacancies and semi-metallic molybdenum dioxide with metallic properties. Due to its unique physical and chemical properties, molybdenum trioxide has been applied in many fields, such as photocatalysis [1,2,3], hole injector for organic solar cells [4,5,6] and high-capacity lithium-ion batteries [7,8,9]. Among the applications of molybdenum oxide, substoichiometric molybdenum oxide has gradually received more attention because of its excellent optical, electrical and catalytic properties. In the field of optoelectronics, MoO_x_ can be used in the antireflection layer of displays [10,11], to improve solar energy conversion efficiency [12,13,14,15] and in new-generation sodium magnesium batteries [16,17,18]. In addition, it can also be used in catalysis [19,20], biomedicine [21], electronic devices [22,23] and other fields.

Although MoO_x_ has excellent properties, little research has been conducted on its thermal stability. As weakly stable oxides, Gunnar Hägg and Arne Magnéli found that Mo_4_O_11_ decomposes at 700–850 °C, and Mo_9_O_26_ converts to Mo_8_O_23_ at 765 °C; it has also been proposed that Mo_4_O_11_ melts into a liquid phase and MoO_2_ at 818 °C, and Mo_9_O_26_ melts into Mo_4_O_11_ and a liquid phase at 780 °C [24]. In recent years, studies on its stability have mainly focused on the usage scenarios of two-dimensional (2D) materials. Jinyoun Cho et al. [25] and Shuangying Cao et al. [26] found that the maximum heat treatment temperature of the MoO_x_ layer in silicon heterojunction solar cells should not exceed 170–200 °C, otherwise a significant redox reaction and metal atom diffusion will occur. The study also found that annealing under argon can effectively improve the thermal stability of MoO_x_ [27,28]. It can also be stacked with high work function metals, e.g., Ni, and subjected to certain heat treatments for higher stability [29]. Hennrik Schmidt et al. [11] found that the original amorphous MoO_x_ films prepared by magnetron sputtering began to transform into crystalline MoO_2_ films after thermal treatment at 350 °C. It can be observed that this substoichiometric molybdenum oxide exists stably only within a specific temperature range, and the thermal stability, decomposition mechanism and phase generated in the decomposition process have not been studied in detail. Therefore, it is necessary to explore its thermal stability and provide a theoretical basis for expanding its application range, such as preparing MoO_x_ ceramic targets, thus producing films with a specific O/Mo ratio.

In this work, the Mo_9_O_26_ and Mo_4_O_11_ substoichiometric molybdenum oxide powders were heated in argon to explore the thermal stability and decomposition behavior of these two powders, and the sintering process was determined according to the thermal stability. Four ceramic bulks, MoO_2.9_, MoO_2.8_, MoO_2.7_ and MoO_2.6_, were prepared by SPS, and the relative density, reflectivity and resistivity were characterized. This work provides useful information for the use of substoichiometric molybdenum oxide in optical and electrical fields.

## 2. Materials and Methods

### 2.1. Preparation of Substoichiometric Molybdenum Oxide Powders

Two powders with main phases of Mo_4_O_11_ (7.47 µm) and Mo_9_O_26_ (5.58 µm) are prepared. The MoO_3_ (99.95%, 2.89 µm) powder is placed into a corundum crucible (80 × 40 × 20 mm^3^) and then into a tubular furnace. The tube furnace is evacuated to vacuum (−0.1 MPa); then, argon is injected at a flow rate of 70 mL/min. The MoO_3_ is heated to 500–600 °C at a heating rate of 5 °C/min in a protective atmosphere. When the preset temperature is reached, the argon gas is turned off, and hydrogen gas is introduced. After holding for a period of time (Mo_9_O_26_ is reduced for 15–45 min, Mo_4_O_11_ is reduced for 45–75 min), the MoO_3_ is slightly reduced by hydrogen to obtain substoichiometric molybdenum oxide. Substoichiometric molybdenum oxide is used for the characterization of Section 2.2 and the mixing and sintering of Section 2.3.

### 2.2. Thermal Stability Tests

The prepared Mo_9_O_26_ and Mo_4_O_11_ powders are subjected to synchronous differential scanning calorimetry (DSC) analysis and thermogravimetric analysis (TGA) on a synchronous thermal analyzer to explore the thermal stability of the oxides. In order to further explore the decomposition behavior and phase evolution of the two oxides at different temperatures, Mo_9_O_26_ and Mo_4_O_11_ are heat-treated in the temperature range of 700–1000 °C and 700–1100 °C for 1 h, respectively, using a tube furnace and corundum crucible (50 × 40 × 20 mm^3^). The isothermal stability test is carried out at an argon flow rate of 70 mL/min. The tubular furnace maintains the same heating rate (10 °C/min) as the synchronous thermal analysis. When the oxide melts at high temperatures and cools to room temperature to become bulky, the bulk is ground into powder using a mortar. Pure phase Mo_9_O_26_ and MoO_3_ (phase pure from XRD analysis; pure Mo_9_O_26_ is prepared by optimizing the hydrogen reduction process of Section 2.1, and MoO_3_ is the same as that used in Section 2.1) are used for heat treatment at 800–900 °C and 900 °C, respectively (conditions consistent with the isothermal thermal stability tests), as supplementary experiments to the isothermal thermal stability test experiments.

### 2.3. Sintered MoO_x_ Ceramics

The substoichiometric molybdenum oxide powders prepared in Section 2.1 are mixed for sintering the ceramic bulks. By adjusting the ratio of substoichiometric molybdenum oxide, the O/Mo atomic ratio of the ceramic bulks can be controlled. In order to distinguish it from the unmixed powder, the mixed powder and ceramic bulk are expressed in the form of MoO_x_. For molybdenum oxide with an O/Mo atomic ratio of 2.9, there is no need to mix other substoichiometric molybdenum oxide to adjust the atomic ratio, so Mo_9_O_26_ powder is directly used as the raw material for sintering MoO_2.9_. Using a v-type mixer, Mo_9_O_26_ (62.3 wt.%), Mo_4_O_11_ (28.3 wt.%) and MoO_2_ (9.4 wt.%) are uniformly mixed to obtain MoO_2.8_ powder. Mo_4_O_11_ (80 wt.%, 70 wt.%) and MoO_2_ (20 wt.%, 30 wt.%) are uniformly mixed to obtain MoO_x_ powder with O/Mo of 2.6 and 2.5. The speed of the v-type mixer is 50 rad/min, and the mixing time is 6 h. Then, the oxide is placed into a graphite mold with an inner diameter of 20 mm. The sintering process is carried out in the LABOX-675F SPS furnace (SINTER LAND INC., Niigata, Japan) at a pressure of 60 MPa for 15 min. The sintering temperature is between 700 and 800 °C, the heating rate is 100 °C/min, and the temperature in the furnace is detected using infrared temperature measuring equipment.

### 2.4. Characterization

The phase evolution and composition after heat treatment were examined by an X-ray diffractometer (XRD, Empyrean Alpha 1, Malvern Panalytical, Alemlo, The Netherlands) equipped with a diffracted beam monochromator using a Cu Kα radiation source. The micromorphology of the powders was analyzed by scanning electron microscope (SEM, Quanta 200 FEG, FEI, Hillsboro, OR, USA). The powders were sprayed with gold for better electronic conductivity. The TG–DSC measurements of the powders were carried out on a synchronous thermal analyzer (STA 449 F3, NETZSCH, Selbu, Germany) with a heating rate of 10 °C/min in argon (gas flow rate 100 mL/min) using alumina crucibles. The reflectivity was tested with a UV–VIS–NIR spectrophotometer (UV-3600, Shimadzu, Kyoto, Japan). The resistivity was measured with a four-point probe (RTS-8, Guangzhou Four Probe Technology Co., Ltd., Guangzhou, China).

## 3. Results and Discussion

### 3.1. Phase Evolution of Substoichiometric Molybdenum Oxide during Heat Treatment

**Figure 1:**
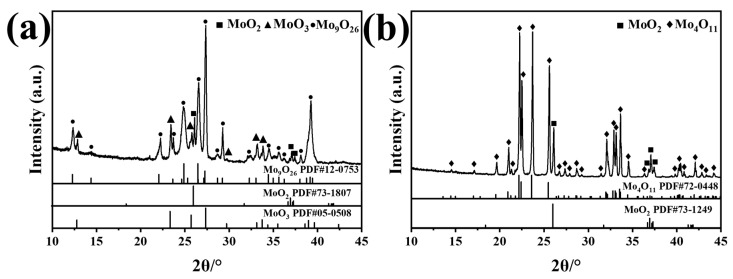
XRD patterns of Mo_9_O_26_ (**a**) and Mo_4_O_11_ (**b**).

The XRD patterns of the Mo_9_O_26_ and Mo_4_O_11_ powders prepared by reducing molybdenum trioxide are shown in Figure 1. Figure 1a shows that the powder is mainly Mo_9_O_26_ (89.4 wt.%) and contains a small amount of MoO_2_ (2.1 wt.%) and MoO_3_ (8.5 wt.%). Figure 1b shows that the powder is dominated by Mo_4_O_11_ (89.7 wt.%), which also contains a small amount of MoO_2_ (10.3 wt.%). The Mo_9_O_26_ and Mo_4_O_11_ powders mentioned below refer to these mixed powders.

**Figure 2:**
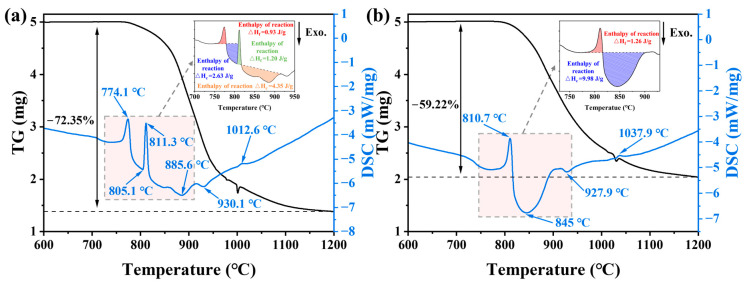
TG–DSC curves recorded during heating of the Mo_9_O_26_ (**a**) and Mo_4_O_11_ (**b**) powders up to 1200 °C at a rate of 10 °C/min in flowing argon.

Simultaneous TG–DSC curves for the Mo_9_O_26_ and Mo_4_O_11_ powders are shown in Figure 2. Figure 2a shows that the DSC curve contains three endothermic peaks at 774.1 °C, 811.3 °C and 1012.6 °C. There are three exothermic peaks at 805.1 °C, 885.6 °C and 930.1 °C, indicating that new phase crystallization may occur at these three temperatures. According to the TG curve, the initial mass reduction occurred at 766.1 °C. At approximately 1000 °C, the TG curve has an obvious anomaly, which corresponds to the endothermic peak at 1012.6 °C (Figure 2a). The final residual mass detected at 1200 °C is 27.7%. The Figure 2b DSC curve contains two endothermic peaks at 810.7 °C and 1037.9 °C. Exothermic peaks appear at 845 °C and 927.9 °C. According to the TG curve, the initial mass reduction occurred at 793.0 °C. At approximately 1030 °C, the TG curve, similar to Mo_9_O_26_, also has an obvious anomaly. This corresponds to the endothermic peak at 1037.9 °C (Figure 2b), which may be due to the violent sublimation [30]. The final residual mass detected at 1200 °C is 40.8%. According to the area method shown in Figure 2, the reaction enthalpy during the heat treatment is indicated.

**Figure 3:**
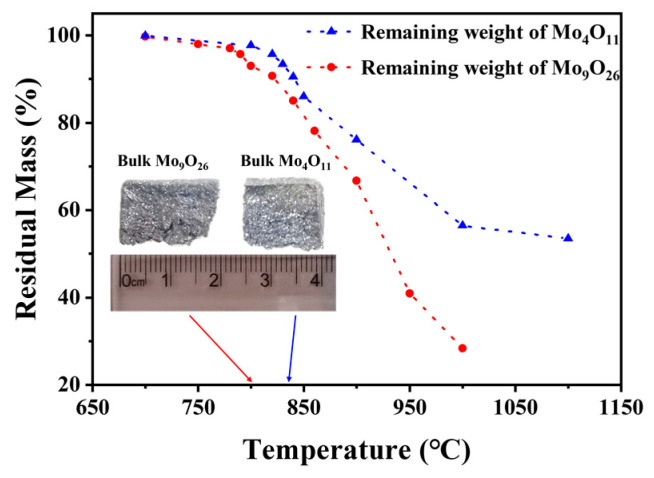
Residual mass of substoichiometric molybdenum oxides after heat treatment.

Mo_9_O_26_ and Mo_4_O_11_ were subjected to isothermal heat treatment with reference to the temperature points obtained by the synchronous thermal analysis. As shown in Figure 3, the mass residual curve of substoichiometric molybdenum oxide after heat treatment is consistent with the TG curve. This same trend shows that the thermal decomposition behavior in the tubular furnace is consistent with that in the synchronous thermal analysis. Notably, as the heat treatment temperature increases, the powders become bulky when cooled to room temperature. The Mo_9_O_26_ powders form a bulk at 800 °C, and the Mo_4_O_11_ powders form a bulk at 840 °C (Figure 3). This is consistent with the judgment in the literature [24] that, above 800 °C, the Mo-O system will be in a liquid state.

**Figure 4:**
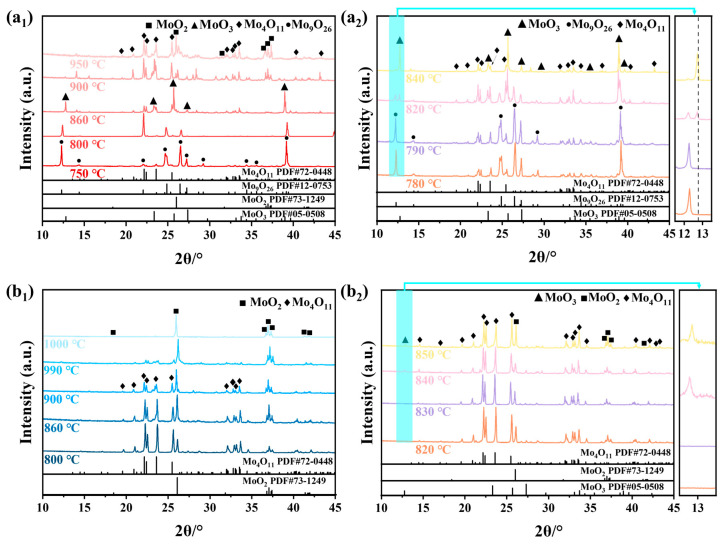
XRD patterns of the Mo_9_O_26_ (**a_1_**,**a_2_**) and Mo_4_O_11_ (**b_1_**,**b_2_**) powders after heat treatment.

Figure 4 shows the XRD spectrums of Mo_9_O_26_ and Mo_4_O_11_ after heat treatment at 750–950 °C and 800–1000 °C, respectively. Table 1 and Table 2 list the phase composition based on Figure 4. It can be observed from Table 1 that Mo_9_O_26_ still exists stably at 790 °C. MoO_3_ and MoO_2_ that originally existed in Mo_9_O_26_ disappeared, and Mo_4_O_11_ phase appeared, which may be the reaction (1):

(1) $$\begin{array}{c} {3\mathrm{MoO}}_{3}{+\mathrm{MoO}}_{2}\;{=\mathrm{Mo}}_{4}O_{11} \end{array}$$

This situation is similar to the method of Jie Dang et al. [31] to prepare Mo_4_O_11_. The slight decrease in mass should be attributed to the sublimation of MoO_3_. At 800 °C, MoO_3_ and Mo_4_O_11_ appear, and the content of Mo_9_O_26_ decreases significantly, indicating that Mo_9_O_26_ begins to fusion, and its enthalpy of fusion is 0.93 J/g. Between 820–840 °C, Mo_9_O_26_ gradually disappears, as shown in Figure 4a_2_. At 860 °C, Mo_9_O_26_ completely disappeared, and only Mo_4_O_11_, MoO_3_ and MoO_2_ three phases existed. New phase MoO_2_ is generated, which corresponds to the exothermic peak at 885.6 °C. At 900–950 °C, as MoO_2_ gradually becomes the main phase, the content of Mo_4_O_11_ decreases, and MoO_3_ has been completely sublimated in this temperature range.

As shown in Table 2, Mo_4_O_11_ is still stable at 830 °C. After heat treatment at 840 °C, the content of Mo_4_O_11_ decreases, and a trace of MoO_3_ appears, indicating that Mo_4_O_11_ begins to fuse at this temperature, and the enthalpy of fusion is 1.26 J/g. With the increasing temperature, Mo_4_O_11_ continues to decompose, MoO_3_ sustains to sublimate, and the content of MoO_2_ gradually increases. Finally, only MoO_2_ exists at 1000 °C. It is worth noting that there are thermodynamically unstable β-MoO_3_ (PDF#47-1320, not marked in Figure 4) and thermodynamically stable α-MoO_3_ (PDF#05-0508) during the decomposition process of substoichiometric molybdenum oxide. There is a trend of β-MoO_3_ to α-MoO_3_ with the increase in temperature. This transition from the thermodynamically unstable phase to the thermodynamically stable phase is accompanied by heat release, which is consistent with the exothermic peak present at approximately 930 °C in Figure 2. From the above results, we can reasonably obtain the thermal stability of Mo_9_O_26_ and Mo_4_O_11_. The former exists stably from room temperature to 790 °C, and the latter exists stably from room temperature to 830 °C.

### 3.2. Decomposition Process of Substoichiometric Molybdenum Oxide during Heat Treatment

**Figure 5:**
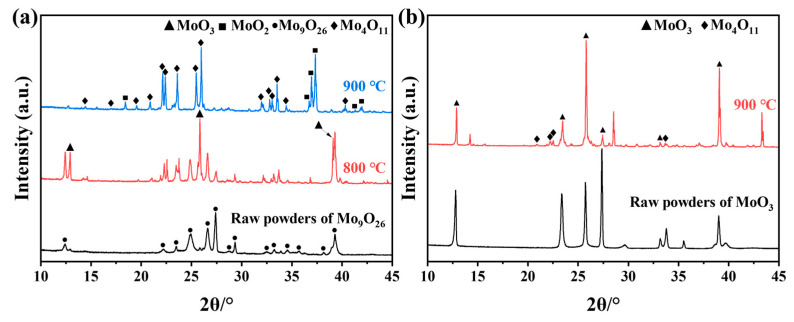
XRD patterns of the Mo_9_O_26_ (**a**) and MoO_3_ (**b**) powders after heat treatment.

In order to exclude the interference of Mo_4_O_11_ generated by Equation (1) during the heat treatment, high-purity Mo_9_O_26_ was heat treated at 800 °C and 900 °C. As can be observed from Figure 5a, Mo_4_O_11_ appeared after heat treatment. As shown in Figure 5b, MoO_3_ forms Mo_4_O_11_ when heat treated at 900 °C, that is, oxygen escape occurs in molybdenum oxide during heat treatment. The unassigned peaks in Figure 5b are Al_2_(MoO_4_)_3_ and Al_2_O_3_ impurities introduced by the alumina crucible.

According to the phase evolution (Figure 4) and the decrease in the O/Mo atomic ratio (Figure 5b), the decomposition process of Mo_9_O_26_ and Mo_4_O_11_ can be summarized as the following reactions:

(2) $$\begin{array}{c} {\mathrm{Mo}}_{9}O_{26}\;{=\mathrm{Mo}}_{4}O_{11}{+5\mathrm{MoO}}_{3} \end{array}$$

(3) $$\begin{array}{c} {2\mathrm{Mo}}_{9}O_{26}\;{=4\mathrm{Mo}}_{4}O_{11}{+2\mathrm{MoO}}_{3\;}{+O}_{2} \end{array}$$

(4) $$\begin{array}{c} {\mathrm{Mo}}_{4}O_{11}\;{=\mathrm{MoO}}_{2}{+3\mathrm{MoO}}_{3} \end{array}$$

(5) $$\begin{array}{c} {2\mathrm{Mo}}_{4}O_{11}\;{=4\mathrm{MoO}}_{2}{+4\mathrm{MoO}}_{3\;}{+O}_{2} \end{array}$$

**Figure 6:**
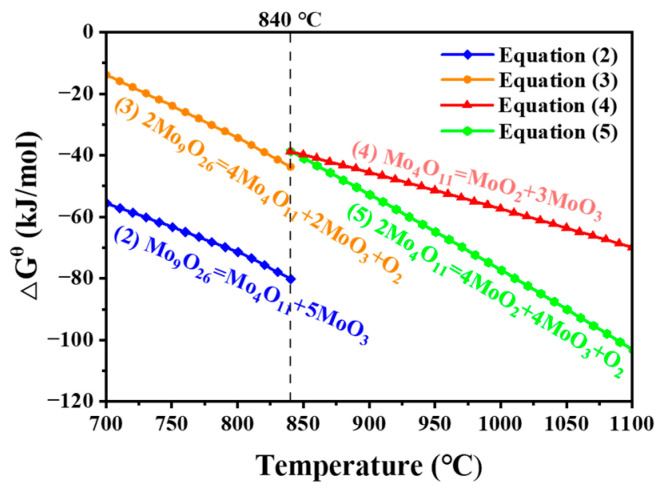
Changes in Gibbs free energy of thermal decomposition of Mo_9_O_26_ and Mo_4_O_11_ with temperature under standard state.

Thermodynamic analysis software HSC6.0 was used to calculate the thermodynamics of the equations. Figure 6 shows the Gibbs free energies as a function of temperature for the four decomposition reactions possible for the two substoichiometric molybdenum oxides in the standard state (101.325 kPa), and it can be observed that all four reactions reach the thermodynamic condition at high temperature.

According to the research results in Section 3.1, only MoO_2_ exists above 1000 °C, the TG curve has significant jitter, and the DSC curve has an endothermic peak; it is reasonable to speculate that Mo_4_O_11_ will sublime rapidly above 1000 °C (Figure 2a,b sublimation 10.1% and 8.9%, respectively). For Mo_4_O_11_, it decomposes below 1000 °C according to Equation (4) or (5) (excluding the sublimated part above 1000 °C), and the final theoretical mass residue is 28.7% or 47.4%, respectively. The actual mass residue is 40.8% (Figure 2b), which is between Equations (4) and (5). This indicates that, during the Mo_4_O_11_ decomposition, both Equations (4) and (5) occur. According to the law of conservation of mass, it can be deduced that 64.4 wt.% of Mo_4_O_11_ decomposes according to Equation (5), and another 35.6 wt.% decomposes according to Equation (4). For Mo_9_O_26_, according to the Equation (2) or (3), the theoretical mass residues are 16.4% or 29.7%, respectively. Therefore, during the decomposition of Mo_9_O_26_, Equations (2) and (3) occur simultaneously with the former accounting for 15.1 wt.%, and the latter accounting for 84.9 wt.%. In summary, most of the mass loss of the two substoichiometric molybdenum oxides come from MoO_3_, and a few are due to the sublimation of Mo_4_O_11_ above 1000 °C.

**Figure 7:**
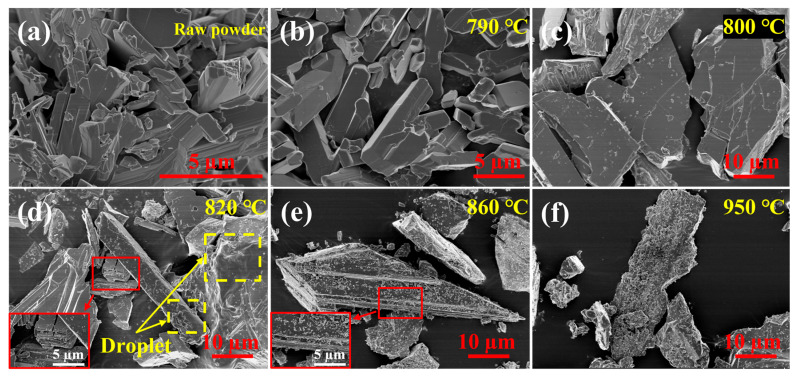
Surface morphologies of the Mo_9_O_26_ raw powders (**a**) and them after heat treated at 790 °C (**b**), 800 °C (**c**), 820 °C (**d**), 860 °C (**e**) and 950 °C (**f**) for 1 h in flowing argon.

**Figure 8:**
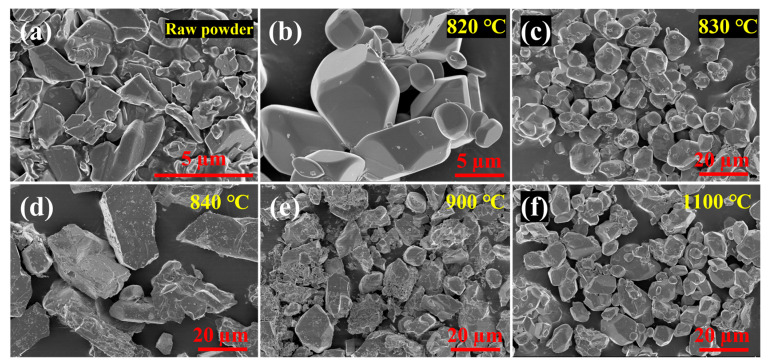
Surface morphologies of the Mo_4_O_11_ raw powders (**a**) and them after heat treated at 820 °C (**b**), 830 °C (**c**), 840 °C (**d**), 900 °C (**e**) and 1100 °C (**f**) for 1 h in flowing argon.

Figure 7 and Figure 8 show the morphology of the Mo_9_O_26_ and Mo_4_O_11_ powders after heat treatment at 790–950 °C and 820–1100 °C, respectively. From Figure 7a,b, it can be found that the layered features of Mo_9_O_26_ disappear after heat treatment. As shown in Figure 7c,d, Mo_9_O_26_ begins to melt between 800–820 °C. The melting phenomenon can be observed in Figure 7d. There are obvious traces of droplets on the powders, and there is no particle adhesion on the surface of the droplets. The droplets are the residues of liquid MoO_3_ that are not volatilized completely. As the temperature continues to increase to 860 °C (Figure 7e), subgrains are found on the powder surface, and the powder again shows distinct layered features (Figure 7d,e). The presence of layered features in Figure 7d,e may be due to the Mo_9_O_26_ of the layered structure [32] with high energy at the interlaminar interface, such that the decomposition reaction occurs here first, and the powder is turned into thinner particles. This transformation is very similar to the crackling core model (CCM). During the temperature increase from 860 °C (Figure 7e) to 950 °C (Figure 7f), the subgrains on the surface of the powder begin to grow and increase significantly, and the plate-like particles are completely covered at 950 °C. From the perspective of phase transition between 860–950 °C, MoO_2_ continues to increase, and Mo_4_O_11_ gradually decreases, so it can be judged that the small particles added on the surface are newly formed MoO_2_ phases. This indicates that the transition from Mo_4_O_11_ to MoO_2_ during heat treatment is an external-to-internal reaction.

As shown in Figure 8a,b, Mo_4_O_11_ grows from an irregular polygonal structure to a regular geometry in which small disk-shaped particles are embedded. This adhesion of different particles may be caused by the low melting point eutectic [33]. At 830 °C (Figure 8c), the Mo_4_O_11_ powder is nearly spherical. Decomposition begins at 840 °C (Figure 8d), and small particles begin to appear on the surface. At 900 °C (Figure 8e), small particles increase and grow significantly and coat on the powder surfaces. From the phase change, it can be observed that, between 840–900 °C, the MoO_2_ content continues to increase, so these gradually increasing small particles are MoO_2_. At 1100 °C (Figure 8f), the powder is completely transformed into polygonal MoO_2_ (the XRD pattern of heat treatment at 1100 °C is consistent with that at 1000 °C, so it is not shown in Figure 4). It can be found that the two kinds of Mo_4_O_11_ are (1) obtained by thermal decomposition of Mo_9_O_26_ at 860 °C and above, and (2) obtained by hydrogen reduction and have the same transformation behavior, which fits the chemical vapour transport (CVT) model from outside to inside; the former is shown in Figure 7e,f, and the latter is shown in Figure 8e,f. The transformation of molybdenum oxide undergoes CCM and CVT, which is different from the view of Werner V. Schulmeyer et al. [34], who think that MoO_2_ is obtained by hydrogen reduction only through the CVT model, which may be because they have not found that Mo_9_O_26_ or the conditions of the two reactions (heat treatment or hydrogen reduction) are different.

**Figure 9:**
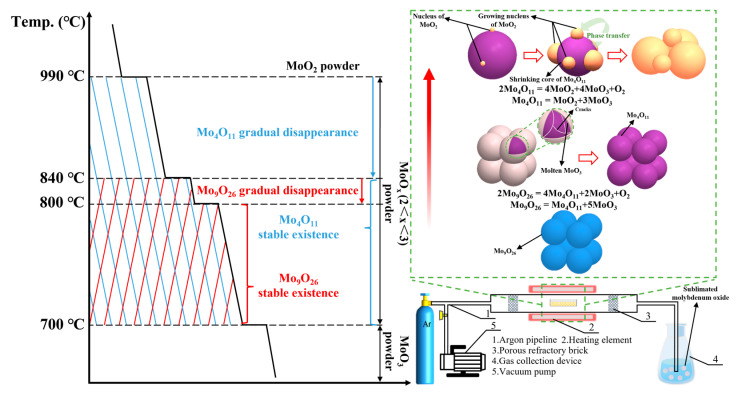
Thermal stability and decomposition mechanism of substoichiometric molybdenum oxides.

The stability and decomposition mechanism of substoichiometric molybdenum oxide are shown in Figure 9. Below 800 °C, Mo_9_O_26_ can exist stably. Then, Mo_9_O_26_ cracks through the CCM at 800 °C and begins to convert to Mo_4_O_11_. Until 840 °C, Mo_9_O_26_ almost decomposes completely. Mo_4_O_11_ is stable below 840 °C and forms MoO_2_ subgrains on the surface by CVT at 840 °C; then, the subgrains increase and grow, and finally replace Mo_4_O_11_ completely from the surface to the interior.

**Figure 10:**
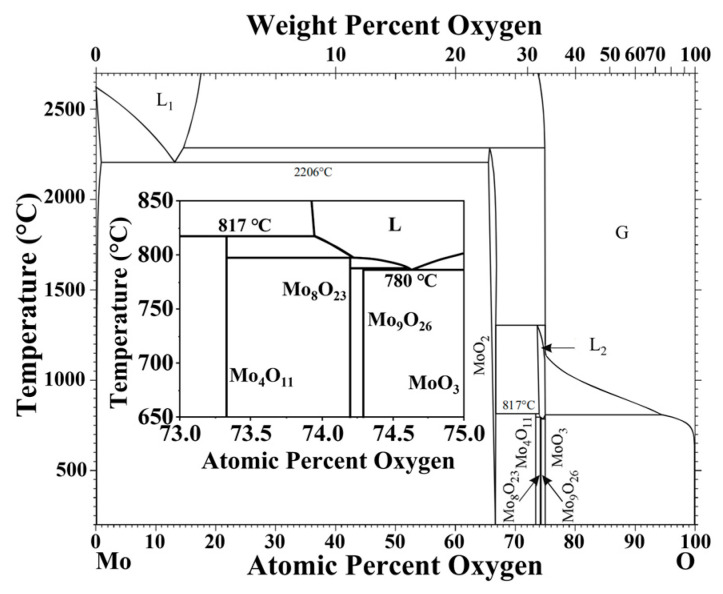
Mo–O binary phase diagram [35].

Comparing the experimental results with the Mo-O phase diagram (Figure 10), we find that the decomposition process of Mo_4_O_11_ is consistent with the phase diagram, transforming into MoO_2_ and MoO_3_ at high temperatures. The decomposition of Mo_9_O_26_ is not consistent with the phase diagram; it is directly decomposed into Mo_4_O_11_ and MoO_3_, and Mo_8_O_23_ does not appear during the decomposition process. The decomposition temperatures of Mo_4_O_11_ and Mo_9_O_26_ phases in the phase diagram are lower than those of the isothermal thermal stability experiments, but they are consistent with the temperature at which the first endothermic peak appears in the DSC curve (Figure 2).

### 3.3. Phase Composition, Optical and Electrical Properties of MoO_x_ Ceramic Bulks

**Figure 11:**
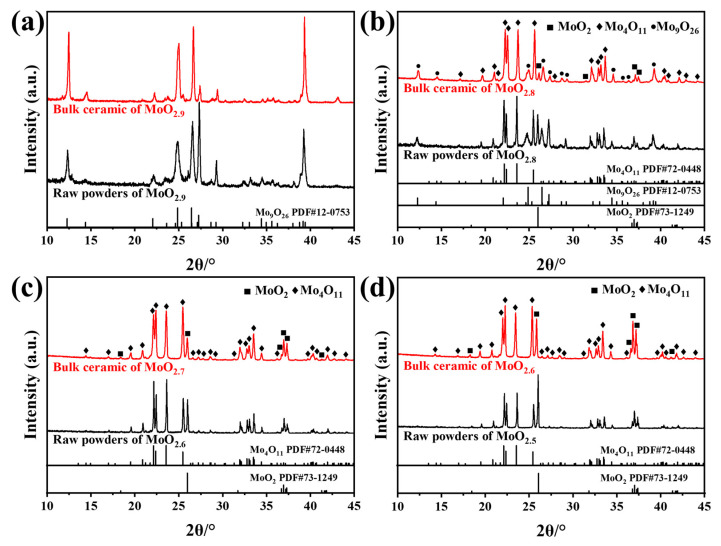
XRD patterns of A (**a**), B (**b**), C (**c**) and D (**d**) before and after sintering.

The Archimedes drainage method was used to determine the relative density of four different x ceramic materials prepared by SPS sintering, which were higher than 96.4%. The phase composition of the ceramic bulk is shown in Figure 11. The phase of MoO_2.9_ (Sample A) and MoO_2.8_ (Sample B) did not change before and after sintering. After sintering with MoO_2.6_ (Sample C) and MoO_2.5_ (Sample D), Mo_4_O_11_ increased by 10 wt.%, and MoO_2_ decreased by 10 wt.%. C and D rose to MoO_2.7_ and MoO_2.6_, respectively, which should be due to the increase in MoO_2_ content that promoted its reaction with the undetected high x (O/Mo atomic ratio, x > 2.75) molybdenum oxide in the raw material, thus producing Mo_4_O_11_.

**Figure 12:**
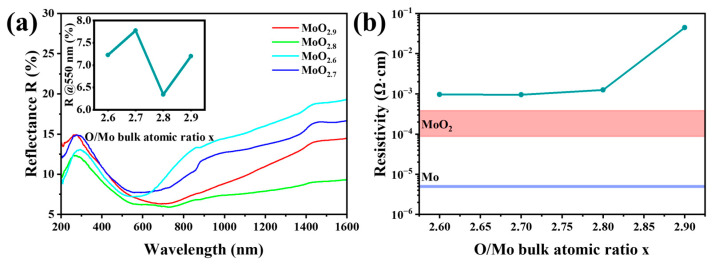
Reflectivity (**a**) and resistivity (**b**) of the MoO_x_ ceramics. The resistivity also includes the reference values of metal Mo and MoO_2_ in the literature [36].

The optical and electrical properties of the MoO_x_ bulks are characterized by reflectivity and resistivity tests. The reflectivity and resistivity are shown in Figure 12. At the most sensitive area of the human eye (550 nm), the reflectivity is between 6.3 to 7.8%, among which the reflectivity of MoO_2.8_ is the lowest at 6.3%. With the decrease in x, the resistivity decreases from 10^−1^ to 10^−3^ Ωcm, and the measured value is close to the value recorded in the literature [36]. From the change in resistivity, MoO_2_ with low resistivity significantly affects the conductivity of the MoO_x_ ceramics. MoO_2.9_ contains only Mo_9_O_26_, which exhibits the highest resistivity. The resistivity of the other three MoO_x_ ceramics decreases significantly with the addition of MoO_2_. Through the analysis of optical and electrical properties, it can be determined that MoO_x_ material is a low reflective conductive oxide material with great application prospects.

## 4. Conclusions

The thermal stability and decomposition mechanism of Mo_9_O_26_ and Mo_4_O_11_ are systematically studied. Under an argon atmosphere, Mo_9_O_26_ has good thermal stability below 790 °C, gradually decomposes into Mo_4_O_11_ and MoO_3_ between 800–840 °C, and the transformation form is similar to the CCM. At 860–950 °C, Mo_4_O_11_ generated by thermal decomposition decomposes into MoO_2_ and MoO_3_. Mo_4_O_11_ has good thermal stability below 830 °C, is decomposed into MoO_2_ and MoO_3_ from 840 °C and completely transforms into MoO_2_ at 1000 °C. This transition is consistent with the CVT model. The decomposition of molybdenum oxide is the process of oxygen loss with the increase in temperature. In a word, during the heat treatment, the transformation of molybdenum oxide undergoes CCM and CVT successively. The relative density of ceramic bulks obtained by SPS sintering can reach 96.4%. All four MoO_x_ have low reflectivity that ranges from 6.3–7.8%, especially MoO_2.8_ at 6.3%. The resistivity of MoO_x_ decreases from 10^−1^ to 10^−3^ Ωcm with the decrease in x.

## Figures and Tables

**Table 1:** Phase compositions of the Mo_9_O_26_ powders after heat treatment.

Heat Treatment Temperature (°C)	Phase Composition
750	Mo_9_O_26_(main), Mo_4_O_11_
780	Mo_9_O_26_(main), Mo_4_O_11_
790	Mo_9_O_26_(main), Mo_4_O_11_
800	Mo_9_O_26_, Mo_4_O_11_, MoO_3_
820	Mo_4_O_11_, MoO_3_, Mo_9_O_26_
840	Mo_4_O_11_, MoO_3_, Mo_9_O_26_(trace)
860	Mo_4_O_11_, MoO_3_, MoO_2_
900	Mo_4_O_11_(main), MoO_3_, MoO_2_
950	MoO_2_(main), Mo_4_O_11_

**Table 2:** Phase compositions of the Mo_4_O_11_ powders after heat treatment.

Heat Treatment Temperature (°C)	Phase Composition
800	Mo_4_O_11_(main), MoO_2_
820	Mo_4_O_11_(main), MoO_2_
830	Mo_4_O_11_(main), MoO_2_
840	Mo_4_O_11_, MoO_2_, MoO_3_(trace)
850	Mo_4_O_11_, MoO_2_, MoO_3_(trace)
860	Mo_4_O_11_, MoO_2_
900	MoO_2_(main), Mo_4_O_11_
990	MoO_2_, Mo_4_O_11_(trace)
1000	MoO_2_

## Data Availability

The data presented in this study are available on request from the corresponding authors.
